# Mn Cluster-Embedded N/F Co-Doped Carbon toward Mild Aqueous Supercapacitors

**DOI:** 10.3390/ma17061417

**Published:** 2024-03-20

**Authors:** Chen Zheng, Xu Han, Fangfang Sun, Yue Zhang, Zihang Huang, Tianyi Ma

**Affiliations:** 1 Institute of Clean Energy Chemistry, Key Laboratory for Green Synthesis and Preparative Chemistry of Advanced Materials of Liaoning Province, College of Chemistry, Liaoning University, Shenyang 110036, China; zc13940417032@163.com (C.Z.); sff108250@163.com (F.S.); yuezhang1229@163.com (Y.Z.); 2 Engineering Laboratory of Advanced Energy Materials, Ningbo Institute of Materials Technology and Engineering, Chinese Academy of Sciences, Ningbo 315201, China; hanxu@nimte.ac.cn; 3 School of Science, RMIT University, Melbourne, VIC 3000, Australia

**Keywords:** Mn cluster, N/F co-doped carbon, neutral electrolyte, supercapacitor, high energy density

## Abstract

Aqueous supercapacitors have occupied a significant position among various types of stationary energy storage equipment, while their widespread application is hindered by the relatively low energy density. Herein, N/F co-doped carbon materials activated by manganese clusters (NCM) are constructed by the straightforward experimental routine. Benefiting from the elevated conductivity structure at the microscopic level, the optimized NCM-0.5 electrodes exhibited a remarkable specific capacitance of 653 F g^−1^ at 0.4 A g^−1^ and exceptional cycling stability (97.39% capacity retention even after 40,000 cycles at the scanning rate of 100 mV s^−1^) in a neutral 5 M LiCl electrolyte. Moreover, we assembled an asymmetric device pairing with a VO_x_ anode (NCM-0.5//VO_x_), which delivered a durable life span of 95% capacity retention over 30,000 cycles and an impressive energy density of 77.9 Wh kg^−1^. This study provides inspiration for transition metal element doping engineering in high-energy storage equipment.

## 1. Introduction

Aqueous supercapacitors have emerged as promising candidates for large-scale stationary energy storage equipment [1,2,3,4]. Nonetheless, their practical deployment is notably constrained by the relatively low energy density [5,6]. Because the electrode material directly determines the electrochemical performance of aqueous supercapacitors, it is an urgent task to investigate high-performance electrode material. At present, intensive research has been conducted in transition metal oxides, hydroxide, and sulfide electrode materials [7,8,9]. Among them, carbon materials stand out as potential electrode materials for supercapacitors, offering the dual advantages of exceptional electrical conductivity and cost-effectiveness [10,11,12]. However, the electric double-layer energy storage mechanism prevents the carbon material application in various scenarios, especially in the field of high-energy storage [13].

Heteroatom doping emerges as an effective strategy to enhance the energy storage capabilities of carbon materials across multiple dimensions. By virtue of the distinct chemical reactivity and hydrophobicity, heteroatom doping obviously elevates the surface adsorption capacity for electrolyte ions. At the same time, the intrinsic electronic structure of carbon materials can be fine-tuned by incorporating heteroatoms with varying electronegativities. As expected, such a modification method changes the electron density and the energy level distribution within the carbon framework, thereby greatly optimizing the carbon material’s electrochemical activity. For example, Qian et al. utilized rapeseed meal as a precursor to develop N and S co-doped carbon material electrodes through pre-activation and carbonization techniques, achieving a specific capacitance of up to 303.4 F g^−1^ at 1 A g^−1^ [14]. Komarneni et al. employed leveraging discarded feathers as the carbon source and KOH as an activator to construct N, O co-doped carbon electrodes, which realized the specific capacitance of 709 F g^−1^ at 0.5 A g^−1^ [15]. Shi et al. explored natural leaves as the carbon source, combined with Mg(NO_3_)_2_ and ZnCl_2_ as activators. The obtained heteroatom-doped porous carbon materials exhibited a specific capacitance of 455.3 F g^−1^ at 1 A g^−1^ [16]. Although heteroatom-doped carbon materials exhibit excellent electrochemical performance, challenges still persist in the following aspects: firstly, there is still an absence of studies in the literature investigating the fundamental mechanism of doping metal ions on the electrochemical performance improvement of carbon materials [17,18,19,20]; secondly, carbon-based electrodes realize the discharging/charging process within acidic or alkaline electrolytes, which aggravate equipment corrosiveness and pose a risk to environmental safety.

In this study, utilizing cost-effective urea, PVDF, and Mn(NO_3_)_2_ as raw materials, we developed N/F co-doped carbon with uniformly distributed Mn through the simplified ball-milling method, which realized high yield and a highly selective synthetic route under mild temperature compared with other physical methods. The abundant cavities within the NCM main host facilitated electrolyte full contact with the electrode. The uniformly dispersed Mn^2+^ cations realized the bi-functionality of high reactivity and the hydrophily. Profiting from the synergistic effects, our optimized electrode NCM-0.5 achieved an outstanding specific capacitance of 653 F g^−1^ at 0.4 A g^−1^ in neutral 5 M LiCl. Impressively, the rate of capacitance retention remained at 74.1% even as the current density increased 10-fold. Moreover, the assembled asymmetric supercapacitors (NCM-0.5//VO_x_) delivered an excellent energy density of 77.9 Wh kg^−1^ with a power density of 56.7 W kg^−1^. The NCM-0.5//VO_x_ also exhibited ultra-long cycle durability with no notable capacity decay even after 30,000 cycles. These findings broaden a new horizon in developing cost-effective, safe, and high-energy aqueous supercapacitors for large-scale energy storage applications.

## 2. Materials and Methods

### 2.1. Materials

Urea (>99%) was purchased from Beijing Kangpu Huiwei Technology Co., Ltd. (Beijing, China). Manganese nitrate tetrahydrate (Mn(NO_3_)_2_·4H_2_O, 98%) and acetone (99.5%) were purchased from Sinopharm Chemical Reagent Co., Ltd. (Shanghai, China). Polyvinylidene fluoride (PVDF), superconducting carbon black (Super-P), and N-methyl pyrrolidone (NVP) were purchased from Shanghai Aladdin Bio-Chem Technology Co., Ltd. (Shanghai, China). Carbon cloth (CC) was purchased from Shanghai HESEN Electric Co., Ltd. (Shanghai, China), and graphite foils manufactured from natural expanded graphite were purchased from SGL Group (Wiesbaden, Germany). All the chemicals were used directly without further purification.

### 2.2. Sample Preparation

A homogenous creamy mixture was prepared by uniformly mixing 16 g PVDF, n mmol Mn(NO_3_)_2_·4H_2_O, and 10 mL acetone after high-energy ball milling for 2 h. Subsequently, 5.6 g of urea was added to the creamy mixture and ball milling was continued for an additional 1 h. The resultant mixture was dried at 60 °C in a vacuum environment for 12 h, and then the two-stage annealing process was conducted. The first stage of the process was annealing at 550 °C for 6 h at a heating rate of 5 °C min^−1^ in an argon atmosphere. This was followed by a heating temperature of up to 900 °C for 1 h at a heating rate of 2 °C min^−1^. These Mn cluster-embedded N/F co-doped carbon nanosheets were designated as NCM-x, where x represented varying concentrations as follows: 0.1, 0.3, 0.5, 0.7, 1. The sample lacking the Mn(NO_3_)_2_·4H_2_O addition and subsequent quenching process was named NC.

A VO_x_ anode was prepared through the following process: Initially, 1 mmol of V_2_O_5_ was dissolved in 15 mL of absolute ethanol and stirred at 25 °C for 30 min. The obtained product was filtered and washed with absolute ethanol and DI 3 times and then dried at 60℃ in a vacuum environment for 12 h.

### 2.3. Sample Characterization

The morphology of the electrodes was characterized by a scanning electron microscope (SEM) equipped with an energy dispersive X-ray spectroscopy (EDS) detector (HITACHI, SU8010, Tokyo, Japan). Transmission electron microscope (TEM) images were carried out by JEM-2010, Tokyo, Japan. The crystal structures of the materials were studied by X-ray diffraction (X’Pert Pro, PANalytical B.V., Almelo, The Netherlands). X-ray photoelectron spectroscopy (XPS) was measured on an XPS spectrometer (ESCALAB 250Xi, Thermo Scientific Escalab, Waltham, MA, USA) with Al-Kα radiation (8.34 Å) as the excitation source. The unpaired electrons of the samples were investigated by Electron Paramagnetic Resonance (Bruker EMXplus, A300, Karlsruhe, Germany). The surface area of the prepared samples was investigated by nitrogen adsorption–desorption measurements (QuantaAutosorb IQ, Hillsboro, OR, USA). The mass loading of active materials was measured by the weight difference of the electrode before and after casting, using semi-microbalance (Sartorius BT25S, Göttingen, Germany) with sensitivity of 0.01 mg.

Electrochemical measurements were conducted by an electrochemical workstation (CHI 760E, Shanghai, China). The electrochemical performance of the prepared samples was tested in a three-electrode cell with SCE as the reference electrode, graphite foil as the counter electrode, and 5 M LiCl as the electrolyte. Mixed NCM-x, super-P, and PVDF in the mass ratio of 8:1:1 in N-methyl pyrrolidone solution. Subsequently, the self-made carbon cloth-based current collector (1.0 × 1.0 cm^2^) was immersed into the abovementioned diluted slurry for 5 min, which ensured the mass loading control at 4.0 mg (±0.3 mg). Electrochemical impedance spectroscopy (EIS) was measured at open-circuit potential with a perturbation of 10 mV and the frequency range from 0.01 Hz to 100 kHz. The electrochemical performance of the NCM-0.5//VO_x_ asymmetric supercapacitor device and pouch device was evaluated by the two-electrode testing system in 5 M LiCl.

The gravimetric capacitance of a single electrode can be calculated based on galvanostatic charge–discharge (GCD) profiles according to Equation (1):

(1) $$C_{m}=\frac{I\times t}{\Delta u\times m}$$

where *C_m_* (F g^−1^) is the gravimetric capacitance, *I* is the discharge current (A), *t* is the discharge time (s), Δ*u* is the potential window (V), and *m* is the mass of active materials (g).

The gravimetric capacitance of an ASC device can be calculated based on GCD profiles according to Equation (2):

(2) $$C_{m}=\frac{I\times t}{U\times m}$$

where *C_m_* (F g^−1^) is the gravimetric capacitance, *I* is the discharge current (A), *t* is the discharge time (s), *U* is the potential window (V), and *m* is the total mass of active materials (g).

(3) $$E_{m}=\frac{1000\;\times \;C_{m}\;\times \;U^{2}}{2\;\times \;3600}$$

(4) $$P_{m}=\frac{3600\times E_{m}}{1000\times t}$$

where *C_m_* (F g^−1^) is the gravimetric capacitance and *U* is the operating voltage (V).

## 3. Results

### 3.1. Material Synthesis and Morphology

**Figure 1:**
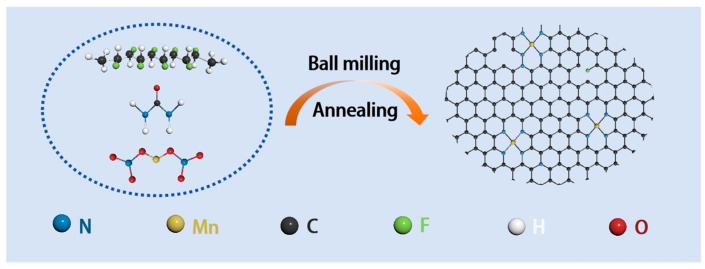
The synthetic route of the NCM-x sample.

Figure 1 briefly elucidates the synthesis route for fabricating the NCM cathode. First of all, the strong electronegative F^−^ in PVDF effectively captured Mn^2+^ in Mn(NO_3_)_2_ during the simple ball milling process, which realized the high dispersion of the Mn active sites on the precursor. After the subsequent annealing process, the urea transformed into g-C_3_N_4_ with larger cavities, and the morphology of the precursor was also regulated into N/F co-doped ultra-thin carbon nanosheets at high temperatures. In this work, the obtained electrode materials displayed optimal electrochemical performance as the feed molar of Mn(NO_3_)_2_ arrived at 0.5 mol (NCM-0.5). For comparison, different feed molar amounts (x = 0, 0.1, 0.3, 0.7, 1) of Mn(NO_3_)_2_ were also doped as reference samples (NCM-x). The sample lacking the Mn(NO_3_)_2_·4H_2_O addition and subsequent quenching process was named NC.

### 3.2. Material Characterization and Analysis

**Figure 2:**
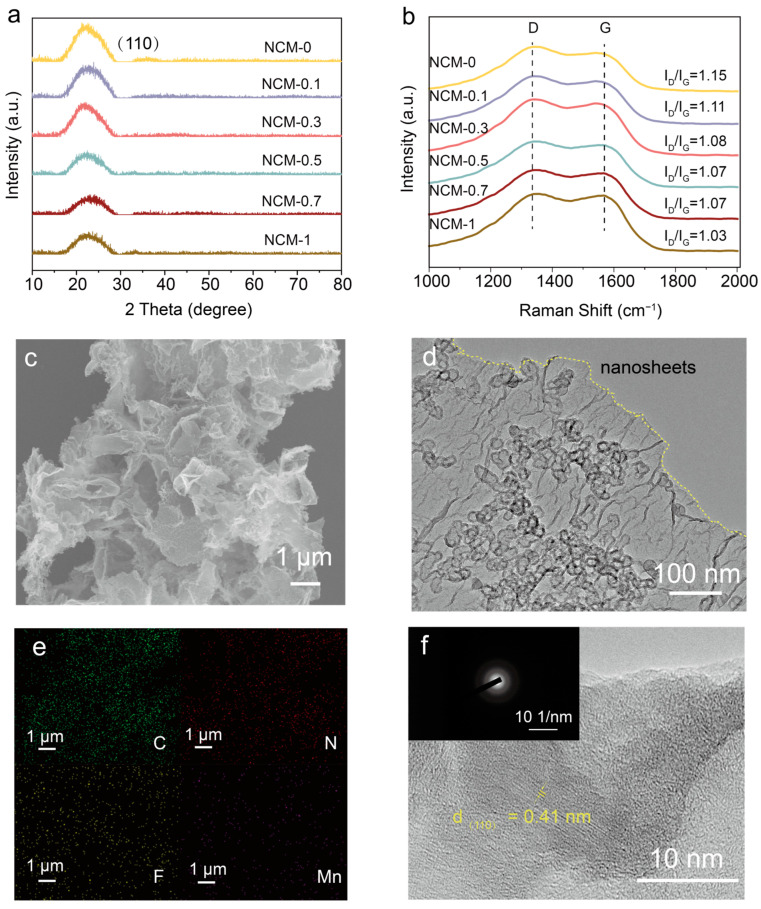
The material characterization of NCM-0.5. (**a**) XRD pattern of NCM-x (x = 0, 0.1, 0.3, 0.5, 0.7, 1); (**b**) Raman spectra of NCM-x (x = 0, 0.1, 0.3, 0.5, 0.7, 1); (**c**) SEM image of NCM-0.5; (**d**) TEM image of NCM-0.5; (**e**) EDS mapping of NCM-0.5; and (**f**) high-resolution TEM image and the SAED pattern of NCM-0.5 (inset).

The crystal structure and composition of the as-synthesized material were characterized by the X-ray diffraction (XRD) pattern (Figure 2a). Notably, the only single-carbon diffraction peak at 2θ = 23° corresponded to the (110) peak of the g-C_3_N_4_ (JCPDS NO.50-0848). According to the Bragg equation, the layer spacing expanded from 0.32 nm to 0.40 nm. There were no other detectable peaks corresponding to metallic Mn or its compounds, confirming the ultra-low content of Mn atoms [21,22]. The extremely similar patterns of different samples demonstrated that the doped Mn atoms did not damage the main structure of the N/F co-doped carbon materials [23]. The Raman spectra were carried out to explore the graphitization degree of NCM-x. Figure 2b shows two characteristic peaks located at 1350 cm^−1^ and 1586 cm^−1^, which were related to D peaks and G peaks. The values of I_D_/I_G_ gradually decreased with an enhancement in Mn(NO_3_)_2_, revealing that the doped Mn atomic cluster successfully improved the conductivity of NCM-x [24]. This phenomenon triggers the disorder of carbon [25].

Scanning electron microscopy (SEM) and transmission electron microscopy (TEM) were carried out to characterize the morphology of NCM-0.5. The comparison between Figure S1a,b indicated that the annealing process converted the original compact morphology into loose nanosheets. As shown in Figure 2c,d, NCM-0.5 exhibited a porous nanosheet structure with adequate cavities, which were conducive to contact with electrolytes. The selected area electron diffraction (SAED) revealed that the doped Mn cluster existed at the atom level rather than the crystalline state. Moreover, energy-dispersive X-ray spectroscopy (EDS) element mapping (Figure 2e and Figure S1c) indicated the uniform distribution of C, N, F, and Mn elements on NCM-0.5. High-resolution TEM (HRTEM) demonstrated that NCM-0.5 possessed a lamellar spacing of 0.41 nm corresponding to (110) peak, which was larger than that of typical graphite (0.335 nm) [26].

**Figure 3:**
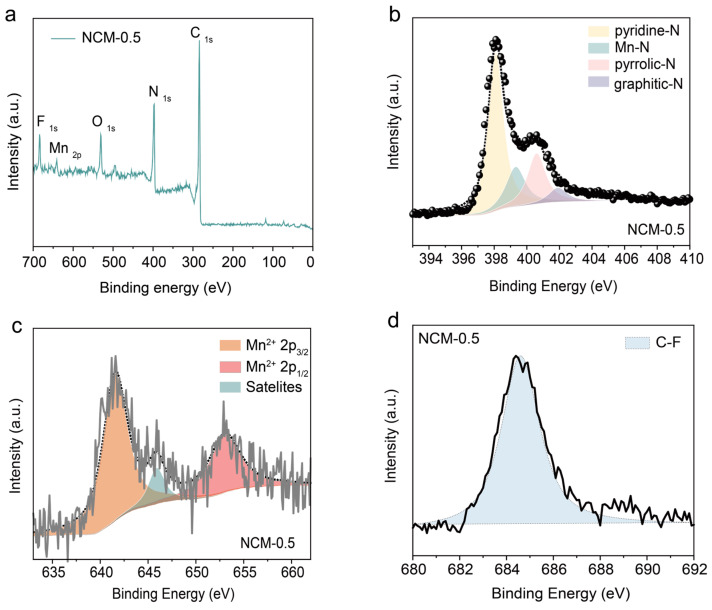
High-resolution (**a**) XPS survey spectra of (**b**) N 1s; (**c**) Mn 2p; and (**d**) F 1s of NCM-0.5.

X-ray photoelectron spectroscopy (XPS) analysis was conducted to obtain insight into the surface composition and the coordination environment of Mn atoms. The survey XPS spectrum prominently featured signals for C, N, O, and Mn elements (Figure 3a). The N 1s core-level spectrum of NCM-0.5 (Figure 3b) exhibited four distinct peaks at 398.1 eV, 399.3 eV, 400.6 eV, and 401.9 eV, corresponding to pyridine-N, Mn-N, pyrrolic-N, and graphitic-N, respectively [27]. However, the pure NCM-0 sample lacked a Mn-N peak (Figure S2a) and the other remaining N 1s spectra were similar to that of NCM-0.5 (Figure S2b–e), which demonstrated that the Mn atoms directly contacted the doped carbon materials. A detailed analysis of the Mn coordination environment was conducted by calculating the percentages of various N species in the synthesized samples, as depicted in Table 1. Intriguingly, the proportion of Mn-N peaks increased with the amount of Mn(NO_3_)_2_ until the mass feed ratio up to 0.5 (first phase); such initial proportion growth can be attributed to the rising feed ratio of Mn(NO_3_)_2_. The proportion of Mn-N peaks was followed by a slight decrease as the mass feed ratio arrived at 0.7 and 1 (second phase), which mainly originated from the agglomeration of the massive Mn atoms and the formation of Mn-Mn metallic bonds. Conversely, the percentage of pyridine-N peaks showed an opposite trend compared with that of Mn-N peaks, suggesting that pyridine-N peaks were more likely to coordinate with Mn atoms. The Mn 2p core-level spectrum (Figure 3c) of NCM-0.5 was deconvoluted into three peaks, which was attributed to Mn^2+^ 2p_3/2_ (641.5 eV), Mn^2+^ 2p_1/2_ (653.1 eV), and a satellite peak (645.9 eV), respectively [28]. This fact indicated that the Mn atomic clusters predominantly exist in the Mn^2+^ state (Figure S3a–d). The high-resolution F 1s spectra of all samples exhibit only one deconvoluted peak for C-F, indicating that the F element does not participate in coordination with Mn atoms (Figure 3d and Figure S4a–e) [29].

### 3.3. Electrode Electrochemical Performance Evaluation

**Figure 4:**
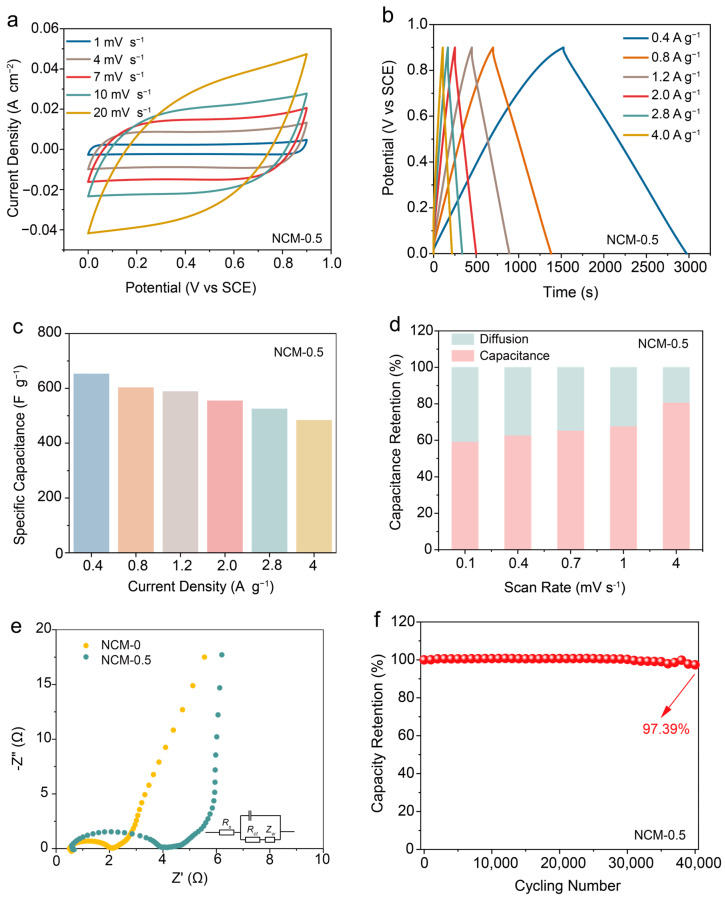
The electrochemical performance and the dynamic process of the electrode. (**a**) CV curves of NCM-0.5 at the scanning rate from 1 mV s^−1^ to 20 mV s^−1^; (**b**) GCD curve of NCM-0.5 at the current density from 0.4 A g^−1^ to 4 A g^−1^; (**c**) the specific capacitance of NCM-0.5 at the current density from 0.4 A g^−1^ to 4 A g^−1^; (**d**) contribution ratio of pseudocapacitive-controlled and diffusion-controlled processes at the scanning rate from 0.1 mV s^−1^ to 4 mV s^−1^; (**e**) EIS spectra of NCM-x (x = 0, 0.5); and (**f**) the long-term cycling ability of NCM-0.5 at the scanning rate of 100 mV s^−1^.

To elucidate the impact of Mn atomic clusters on electrochemical behavior, we evaluated all samples in a three-electrode system. The cycle voltammetry (CV) curves were conducted at a potential window from 0 to 0.9 V. The CV curves of pristine NCM-0 (Figure S5) at the scanning rates of 10 mV s^−1^ demonstrated significant polarization and impeded electron transport paths, which were contrasted with the CV curves of the Mn cluster-anchored NCM-x. Notably, the CV curves of NMC-0.5 (Figure 4a) exhibited the most defined rectangular shape and the highest peak current, revealing enhanced capacitance characteristics. Except for NCM-0 (Figure S6), the galvanostatic discharge/charge (GCD) curve of NCM-0.5 exhibited a clearly symmetric isosceles triangle (Figure 4b), which was similar to the GCD curves of other Mn-doped samples. These observations suggested that Mn atomic clusters substantially optimized the energy storage properties of carbon-based materials through the following aspects: firstly, the dispersed transition metal Mn atomic clusters offered additional electronic states, facilitating electron exchange between electrode materials and electrolytes; secondly, the incorporation of Mn atom clusters induced an uneven charge distribution on the carbon material, leading to localized polarization effects. Additionally, specific capacitances of the prepared electrodes at various current densities are presented in Figure 4c. Remarkably, NCM-0.5 achieved a superior specific capacitance of 653 F g^−1^ at 0.4 A g^−1^, which was approximately 2.65 times that of pure NC under the same current density (Figure S7). In addition, the rate of capacitance retention still retained at 74.1% as the current density increased from 0.4 A g^−1^ to 4 A g^−1^. Such outstanding electrochemical performance exceeded most of the reported state-of-the-art carbon-based electrodes (Table S1). It is worth mention that the specific capacitance did not exhibit a linear correlation with an increasing ratio of the Mn atomic cluster but rather followed a typical volcano plot. This phenomenon demonstrated that the Mn atomic cluster provided more active sites within the appropriate range, while the higher Mn content initiated the aggregation resulting in “dead points” within the optimized electrodes [30]. Hence, we came to the conclusion that the doped Mn atomic cluster plays an essential part in the electrochemical performance of NCM-x.

To further analyze the charge storage mechanism of different samples, we collected CV curves of NC and NCM-0.5 at various scanning rates and calculated the contribution ratio of pseudocapacitive-controlled and diffusion-controlled process based on Dunn’s method [31]:

(5) $$i=k_{1}v+k_{2}v^{1/2}$$

where *k*_1_*v* and *k*_2_*v*^1/2^ represent the pseudocapacitive-controlled and diffusion-controlled processes, respectively. According to the calculation results, NCM-0.5 delivered a higher capacitance contribution of 68.1%. Remarkably, Figure 4d shows the different contribution ratios of the pseudocapacitive-controlled process at various scan rates for NCM-0.5. Such calculation results suggested that the Mn cluster-anchored carbon-based material exhibited superior rate performance and durable cycling stability even under elevated current densities. In order to investigate the transport mechanism of electrolyte ions within electrode materials, we conducted electrochemical impedance spectroscopy (EIS) within a frequency range from 0.01 Hz to 100 kHz. NCM-0.5 demonstrated a steeper slope than that of NC in the low-frequency domain (Figure 4e), which indicated pronounced capacitive behavior. However, NC exhibited an equivalent series resistance (*Rs*) and charge transfer resistance (*Rct*) of 0.52 Ω and 1.47 Ω, which were obviously lower than that of NCM-0.5 (0.59 Ω and 3.72 Ω). Such an intriguing phenomenon was mainly ascribed to microstructural integration with manganese clusters and the carbon matrix, which increased electron scattering at these cluster sites and elevated transport resistance [32]. Nonetheless, this phenomenon does not overshadow the remarkable improvement in the electrochemical performance of the NMC-0.5 samples. To further verify the long-term stability of the NCM-0.5 electrode materials, we tested NCM-0.5 at a scanning speed of 100 mV s^−1^. As shown in Figure 4f, the specific capacitance retention of the NCM-0.5 electrodes was maintained at 97.39% even after undergoing 40,000 cycles.

### 3.4. Design of the Aqueous Asymmetric Supercapacitor

**Figure 5:**
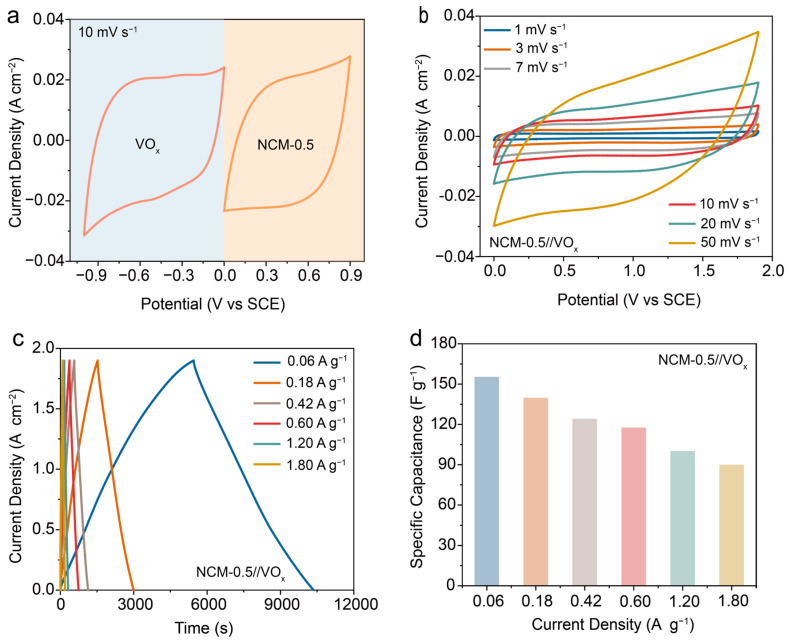

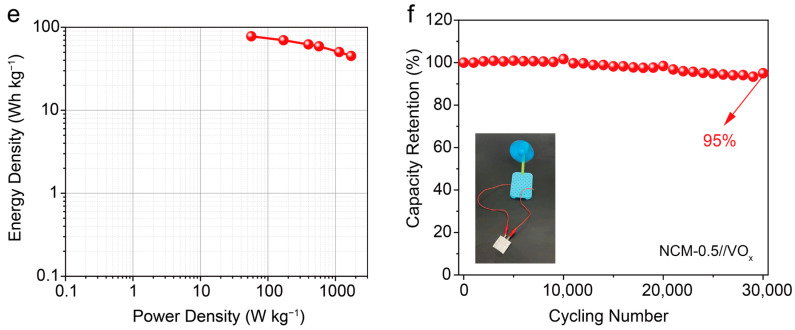
The electrochemical performance of the asymmetric supercapacitor. (**a**) CV curves of the NCM-0.5 cathode and the VO_x_ anode at a scanning rate of 10 mV s^−1^; (**b**) CV curves of NCM-0.5//VO_x_ at the scanning rate from 1 mV s^−1^ to 50 mV s^−1^; (**c**) GCD profiles of NCM-0.5//VO_x_ at the current density from 0.06 A g^−1^ to 1.8 A g^−1^; (**d**) specific capacitance of NCM-0.5//VO_x_ at the current density from 0.06 A g^−1^ to 1.8 A g^−1^; (**e**) Ragone plot of NCM-0.5//VO_x_; and (**f**) the long-term cycling ability of NCM-0.5//VO_x_ at the scanning rate of 100 mV s^−1^ and NCM-0.5//VO_x_ continuously provided power to the fan (inset).

In order to evaluate the practicality of the NCM-0.5 electrode, we designed an asymmetric supercapacitor with NCM-0.5 as the cathode, VO_x_ as the anode, and 5 M LiCl as the electrolyte. The CV curves of the NCM-0.5 cathode (0–0.9 V) and the VO_x_ anode (−1–0 V) at a scanning rate of 10 mV s^−1^ are presented in Figure 5a, which achieved a stable voltage window up to 1.9 V. As illustrated in Figure 5b,c, the CV and GCD curves for the NCM-0.5//VO_x_ device were performed under a voltage window of 1.9 V. As the scan rate increased from 1 mV s^−1^ to 50 mV s^−1^, the CV curve of the NCM-0.5//VO_x_ device was not significantly distorted, which revealed an exceptional fast discharging/charging behavior. Meanwhile, the isosceles triangle-shaped GCD curve at various current densities also confirmed the preferable rate performance and coulombic efficiency. Furthermore, the designed NCM-0.5//VO_x_ device exhibited an excellent specific capacitance of 155.4 F g^−1^ at 0.06 A g^−1^ and maintained 89.7 F g^−1^ at 1.8 A g^−1^ (Figure 5d). Energy density and power density are practical parameters for evaluating supercapacitor performance. Relying on the excellent specific capacitance and wide voltage window, the designed device delivered an energy density of 77.9 Wh kg^−1^ at 56.7 W kg^−1^, which transcended most of the reported state-of-the-art carbon-based devices (Figure 5e, Table 2). In addition, we also tested the long-term cycling stability of the NCM-0.5//VO_x_ devices at a scanning speed of 100 mV s^−1^. As depicted in Figure 5f, the capacity retention of the NCM-0.5//VO_x_ devices arrived at 95% even after 30,000 cycles, which demonstrated the tremendous application potential among various aqueous supercapacitors.

## 4. Conclusions

Herein, exploiting PVDF, urea, and Mn(NO_3_)_2_ as raw materials, we successfully synthesized Mn cluster-embedded N/F co-doped carbon through the environmentally friendly ball-milling method. This process effectively introduced Mn active sites and heteroatomic N/F. At the same time, the porous nanosheet structure with generous cavities was also successfully constructed. Benefiting from the above synergistic effect, the optimized NCM-0.5 electrode delivered an outstanding specific capacitance of 653 F g^−1^ at 0.4 A g^−1^ and an exceptional rate capability retention of 74.1% as the current density increased from 0.4 A g^−1^ and 4 A g^−1^. Moreover, the NCM-0.5 electrode also exhibited ultra-long cycle durability with negligible capacity fading even after 40,000 cycles. Additionally, we fabricated an aqueous asymmetric device by NCM-0.5 cathode pairing with VO_x_ as the anode. The obtained NCM-0.5//VO_x_ achieved an energy density of 77.9 Wh kg^−1^ at 56.7 W kg^−1^, which surpassed most current state-of-the-art devices. This work has effectively improved the electrochemical performance of carbon-based materials in supercapacitors, marking the possibility of future large-scale industrial applications.

## Figures and Tables

**Table 1:** The proportion of different forms of N in the various samples.

Sample	Pyridine-N	Mn-N	Pyrrolic-N	Graphitic-N
NCM-0	60.7%	0%	17.9%	21.4%
NCM-0.1	58.5%	8.6%	22.7%	10.2%
NCM-0.3	57.0%	13.8%	19.4%	9.9%
NCM-0.5	55.2%	17.8%	21.0%	6.0%
NCM-0.7	61.4%	14.8%	16.1%	7.8%
NCM-1	63.3%	11.8%	17.0%	7.9%

**Table 2:** Summary of the energy density and power density of carbon-based devices.

Sample	Energy Density (Wh kg^−1^)	Power Density (W kg^−1^)	Ref.
g-C_3_N_4_/rGO@NiCo_2_S_4_//AC	66	751	[33]
Ni-Co-P@C/Ni-B//HP-N-CFs	68.3	985	[34]
FeNiCoTe//AC	62.9	806.4	[35]
NiV_2_Se_4_-Ag//AC	77	749	[36]
c-PAN@MCA_0.25_//c-PAN@MCA_0.25_	11.4	344	[37]
Fe_2_N@Fe_3_O_4_/NrGO//Ni_2_O/rGO	28.6	825	[38]
rGO/NiMnCo-OH//AC	66.9	800	[39]
Co_1-x_S/HCoO_2_-1@Fe_3_C/PCNFs//Fe_2_O_3_/NPC@PCNFs	65.68	752.7	[40]
NiSe/g-C_3_N_4_//AC	52.5	1488	[41]

## Data Availability

The data presented in this study are available upon request from the corresponding author.

## Supplementary Materials

The following supporting information can be downloaded at: https://www.mdpi.com/article/10.3390/ma17061417/s1, Figure S1: SEM images and EDS spectra; Figure S2: High-resolution XPS spectrum of N 1s; Figure S3: High-resolution XPS spectrum of Mn 2p; Figure S4: High-resolution XPS spectrum of F 1s; Figure S5: CV profiles of NCM-x; Figure S6: GCD profiles of NCM-x; Figure S7: The specific capacitance of NCM-x; Table S1: Comparison of the specific capacitance [42,43,44,45,46,47,48,49,50,51,52,53,54,55,56,57,58,59,60,61,62,63,64,65,66].
