# Supplementary material for: Mn Cluster-Embedded N/F Co-Doped Carbon toward Mild Aqueous Supercapacitors
Source: Materials (Basel). 2024 Mar 20;17(6):1417. doi: 10.3390/ma17061417 (PMC10971931; doi:10.3390/ma17061417)
Supplement: Supplementary file 1 [file materials-17-01417-s001.zip › materials-2888391-supplementary.pdf]

Supporting Information

Mn Cluster-Embedded N/F Co-Doped Carbon toward Mild Aqueous Supercapacitors

Chen Zheng [1,†], Xu Han [2,†], Fangfang Sun [1], Yue Zhang [1], Zihang Huang [1,*] and Tianyi Ma [3,*]

[1] Institute of Clean Energy Chemistry, Key Laboratory for Green Synthesis and Preparative Chemistry of Advanced Materials of Liaoning Province, College of Chemistry, Liaoning University,
Shenyang 110036, China; zc13940417032@163.com (C.Z.); sff108250@163.com (F.S.); yuezhang1229@163.com (Y.Z.)
[2] Engineering Laboratory of Advanced Energy Materials, Ningbo Institute of Materials Technology and
Engineering, Chinese Academy of Sciences, Ningbo 315201, China; hanxu@nimte.ac.cn
[3] School of Science, RMIT University, Melbourne, VIC 3000, Australia
* Correspondence: huangzihang@lnu.edu.cn (Z.H.); tianyi.ma@rmit.edu.au (T.M.)
† These authors contributed equally to this work.

Supplementary Figures and Tables

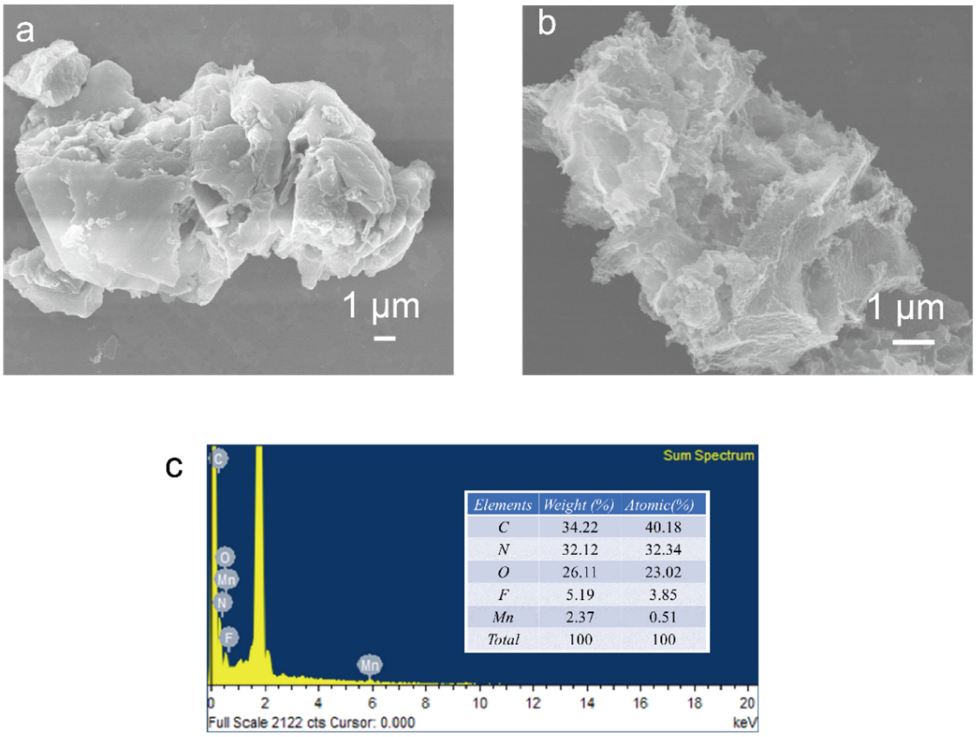

Figure S1. SEM image of (**a**) the NC; (**b**) the NCM-0; (**c**) EDS spectra collected for NCM-0.5 sample.

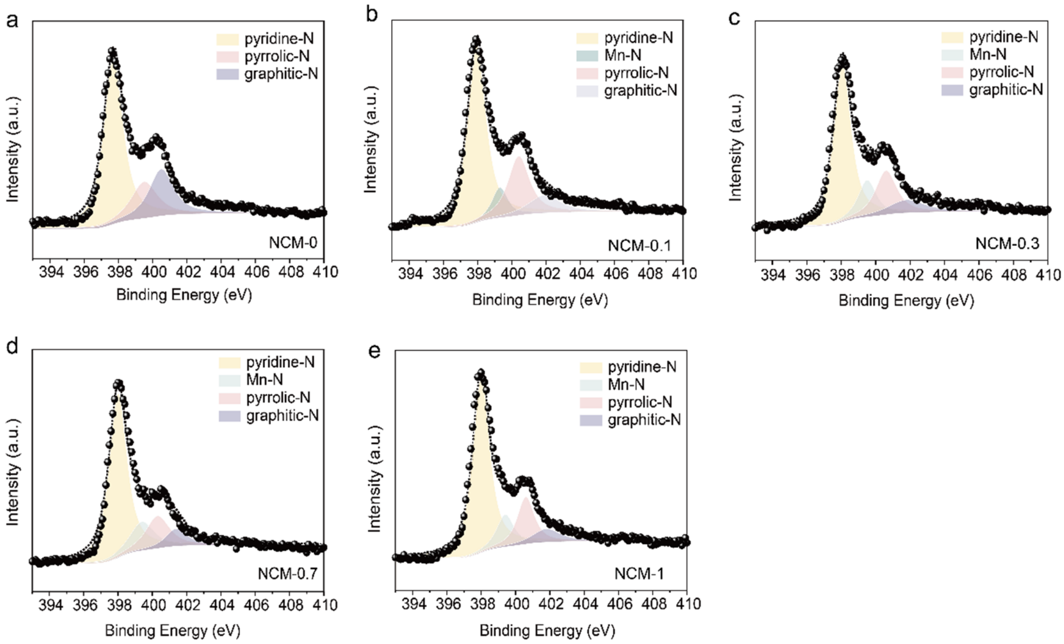

Figure S2. High resolution XPS spectrums of N 1s of (**a**) NCM-0; (**b**) NCM-0.1; (**c**) NCM-0.3; (**d**) NCM-0.7; (**e**) NCM-1.

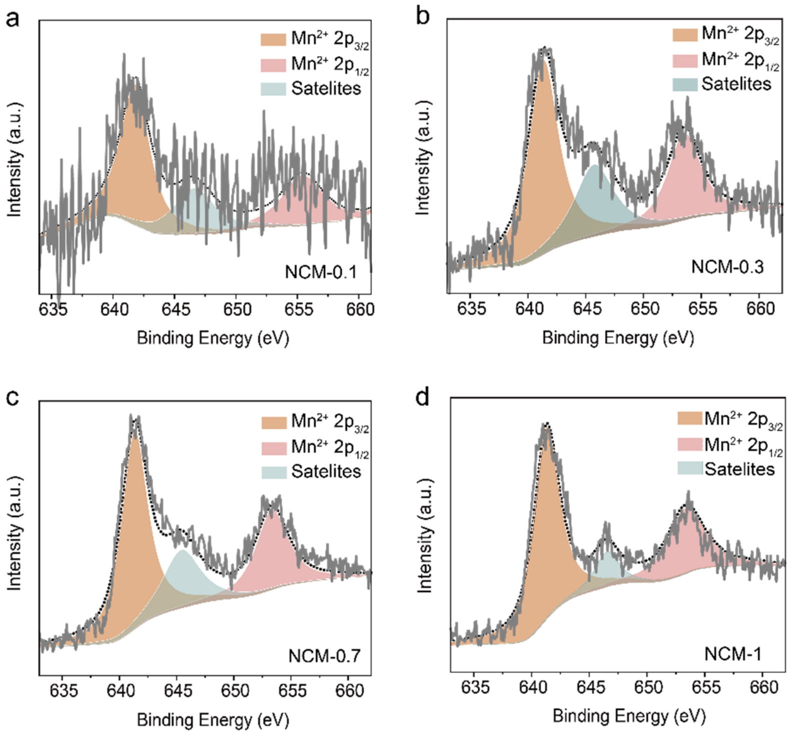

Figure S3. High resolution XPS spectrums of Mn 2p of (**a**) NCM-0.1; (**b**) NCM-0.3; (**c**) NCM-0.7; (**d**) NCM-1.

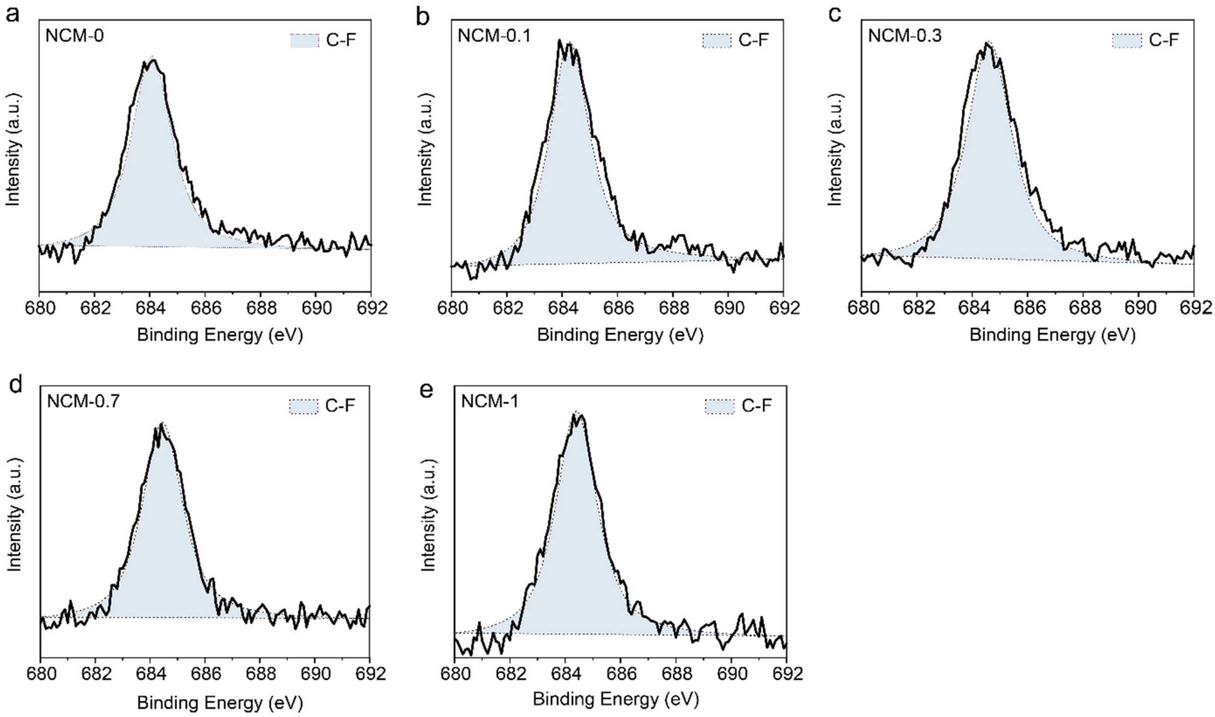

Figure S4. High resolution XPS spectrums of F 1s of (**a**) NCM-0; (**b**) NCM-0.1; (**c**) NCM-0.3; (**d**) NCM-0.7; (**e**) NCM-1.

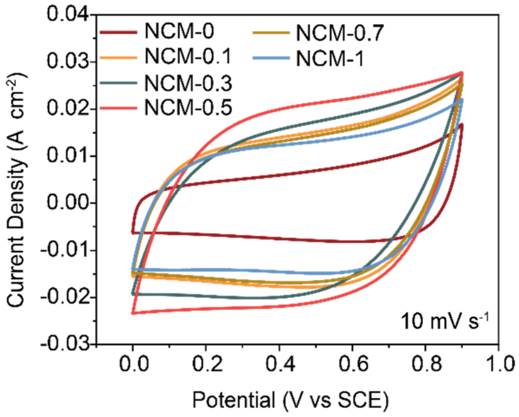

Figure S5. CV curves of NCM-x (x=0, 0.1, 0.3, 0.5, 0.7, 1) at the scanning rate of 10 mV s^{-1}.

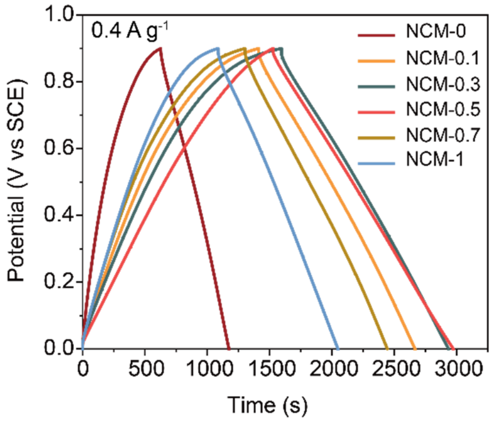

Figure S6 GCD profiles of the NCM-x (x=0, 0.1, 0.3, 0.5, 0.7, 1) at the current density of 0.4 A g^{-1}.

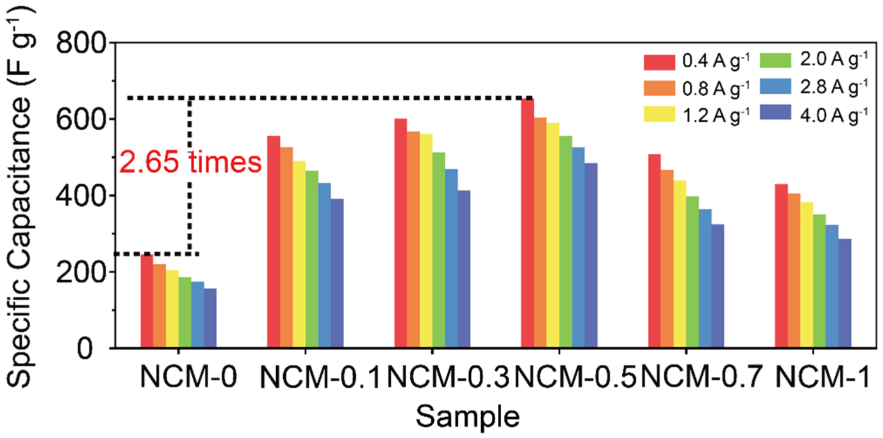

Figure S7 the specific capacitance of the NCM-x (x=0, 0.1, 0.3, 0.5, 0.7, 1) at

various current densities.

Table S1. Comparison of specific capacitance, rate capability and cycling stability of NCM-0.5 with recently reported carbon-based electrodes.

Electrode	Maximum Specific Capacitance	Minimum Specific Capacitance	Rate Capability	Capacitance Retention
HPC-700[1]	412.5 F g^{-1} @ 0.5 A g^{-1}	253 F g^{-1} @ 50 A g^{-1}	61.3%	99.6% (10000)
NIPCG[2]	673 F g^{-1} @ 1 A g^{-1}	481 F g^{-1} @ 10 A g^{-1}	71.5%	95% (1000)
B/N-CNS[3]	423 F g^{-1} @ 0.2 A g^{-1}	125 F g^{-1} @ 50 A g^{-1}	29.5%	100% (30000)
pCA-KOH-700[4]	467 F g^{-1} @ 1 A g^{-1}	330 F g^{-1} @ 10 A g^{-1}	70.6%	85.7% (10000)
HPC-700[5]	330 F g^{-1} @ 1 A g^{-1}	221 F g^{-1} @ 20 A g^{-1}	66.9%	90.5% (10000)
NS-PCM-1000[6]	461.5 F g^{-1} @ 0.1 A g^{-1}	187 F g^{-1} @ 10 A g^{-1}	40.5%	90% (23000)
NCNF2-900[7]	224 F g^{-1} @ 0.5 A g^{-1}	149 F g^{-1} @ 10 A g^{-1}	66.5%	97% (10000)
N/S-HCSs[8]	280 F g^{-1} @ 1 A g^{-1}	134 F g^{-1} @ 50 A g^{-1}	47.8%	94.4% (10000)
AHPC[9]	576 F g^{-1} @ 1 A g^{-1}	460.8 F g^{-1} @ 10 A g^{-1}	80%	95% (10000)
900N-Ti_2CT_x[10]	327 F g^{-1} @ 1 A g^{-1}	282 F g^{-1} @ 10 A g^{-1}	86.2%	96.2% (5000)
MAC-N-0.5[11]	385 F g^{-1} @ 0.2 A g^{-1}	332 F g^{-1} @ 20 A g^{-1}	86.3%	100% (10000)
NOPC-2[12]	527 F g^{-1} @ 1 A g^{-1}	246 F g^{-1} @ 200 A g^{-1}	46.7%	94.3% (20000)
N-GQD@cZIF-8/CNT[13]	540 F g^{-1} @ 0.5 A g^{-1}	332.1 F g^{-1} @ 20 A g^{-1}	61.5%	90.9% (8000)
HHCF[14]	361 F g^{-1} @ 1 A g^{-1}	182 F g^{-1} @ 100 A g^{-1}	50.4%	95.8% (10000)
NPCN[15]	267 F g^{-1} @ 1 A g^{-1}	145 F g^{-1} @ 50 A g^{-1}	54%	_____
FCL700[16]	505.4 F g^{-1} @ 0.1 A g^{-1}	216.1 F g^{-1} @ 20 A g^{-1}	42.7%	70.3% (2000)
KNOSC[17]	402.5 F g^{-1} @ 1 A g^{-1}	308.5 F g^{-1} @ 100 A g^{-1}	76.6%	90% (30000)

2D CoSNC[18]	360.1 F g^{-1} @ 1.5 A g^{-1}	204.5 F g^{-1} @ 30 A g^{-1}	56.8%	90% (2000)
N-CNFs-900[19]	202 F g^{-1} @ 1 A g^{-1}	164 F g^{-1} @ 30 A g^{-1}	81.2%	97% (3000)
NCA-800[20]	300 F g^{-1} @ 0.5 A g^{-1}	242 F g^{-1} @ 10 A g^{-1}	80.6%	98% (5000)
PCNS-6[21]	470 F g^{-1} @ 1 A g^{-1}	338 F g^{-1} @ 20 A g^{-1}	72%	98% (50000)
2D-HPCs[22]	250 F g^{-1} @ 0.5 A g^{-1}	185 F g^{-1} @ 10 A g^{-1}	74%	96% (10000)
ZIF-8/ZTP[23]	341.2 F g^{-1} @ 0.1 A g^{-1}	181.3 F g^{-1} @ 10 A g^{-1}	53.1%	91.5% (50000)
TCNQ-CTF-800[24]	383 F g^{-1} @ 0.2 A g^{-1}	197 F g^{-1} @ 10 A g^{-1}	51.4%	100% (10000)
MPC-800[25]	430 F g^{-1} @ 0.5 A g^{-1}	327 F g^{-1} @ 10 A g^{-1}	76.0%	100% (10000)
**NCM-0.5**	**653 F g^{-1} @ 0.4 A g^{-1}**	**484 F g^{-1} @ 4 A g^{-1}**	**74.1%**	**40000 (97.4%)**

References

[1] Wang, Y.; Liu, R.; Tian, Y.; Sun, Z.; Huang, Z.; Wu, X.; Li, B. Heteroatoms-doped hierarchical porous carbon derived from chitin for flexible all-solid-state symmetric supercapacitors. *Chemical Engineering Journal*, **2019**, *384*,123263.

[2] Chen, Z.; Zhao, S.; Zhao, H.; Zou, Y.; Yu, C.; Zhong, W. Nitrogen-doped interpenetrating porous carbon/graphene networks for supercapacitor applications. *Chemical Engineering Journal*, **2021**, *409*, 127891.

[3] Hao, J.; Wang. J.; Qin; S.; Liu, D.; Li, Y. B/N co-doped carbon nanosphere frameworks as high-performance electrodes for supercapacitors. *Journal of Materials Chemistry A*, **2018**, *6*,8053-8058.

[4] Wei, X.; Wan, S.; Gao, S. Self-assembly-template engineering nitrogen-doped carbon aerogels for high-rate supercapacitors. *Nano Energy*, **2016**, *28*, 206-215.

[5] Qian, X.; Miao, L.; Jiang. J.; Ping, G.; Xiong, W.; Lv, Y.; Liu, Y.; Gan, L.; Zhu, D.; Liu, M. Hydrangea-like N/O codoped porous carbons for high-energy supercapacitors. *Chemical Engineering Journal*, **2020**, *388*, 124208.

[6] Lu, H.; Yang, C.; Chen, J.; Li, H.; Wang, J.; Wang, S. Tailoring Hierarchically

Porous Nitrogen-, Sulfur-Codoped Carbon for High-Performance Supercapacitors and Oxygen Reduction. *Small*, **2020**, *16*, 190584.

[7] Chen, H.; Liu, T.; Mou, J.; Zhang, W.; Jiang, Z.; Liu, J.; Huang, J.; Liu, M. Free-standing N-self-doped carbon nanofiber aerogels for high-performance all-solid-state supercapacitors. *Nano Energy*, **2019**, *63*, 103836.

[8] Miao, L.; Zhu, D.; Liu, M.; Duan, H.; Wang, Z.; Lv, Y.; Gan, Y. Cooking carbon with protic salt: Nitrogen and sulfur self-doped porous carbon nanosheets for supercapacitors. *Chemical Engineering Journal*, **2018**, *347*, 233-242.

[9] Zhao, G.; Chen. C.; Yu. D.; Sun, L.; Yang, C.; Zhang, H.; Sun, Y.; Besenbacher, F.; Yu, M. One-Step Production of O-N-S Co-Doped Three-Dimensional Hierarchical Porous Carbons for High-Performance Supercapacitors. *Nano Energy*, **2018**, *47*, 547-555.

[10] Yoon, Y.; Lee, M.; Kim, S. K.; Bae, G.; Song, W.; Sung, M.; Lim, J.; Lee, S. S.; Zyung. T.; An, K. S. A Strategy for Synthesis of Carbon Nitride Induced Chemically Doped 2D MXene for High-Performance Supercapacitor Electrodes. *Advanced Energy Materials*, **2018**, *8*,1703173.

[11] Zhou, J.; Hou, L.; Lian, J.; Cheng, W.; Wang, D.; Gou, H.; Gao, F. Nitrogen-doped highly dense but porous carbon microspheres with ultrahigh volumetric capacitance and rate capability for supercapacitors. *Journal of Materials Chemistry A*, **2019**, *7*, 476-485.

[12] Hou, L.; Yang, W.; Li, Y.; Wang, P.; Jiang, B.; Xu, C.; Zhang, C.; Huang, G.; Yang, F.; Li, Y. Dual-template endowing N, O co-doped hierarchically porous carbon from potassium citrate with high capacitance and rate capability for supercapacitors. *Chemical Engineering Journal*, **2021**, *417*, 129289.

[13] Li, Z.; Liu, X.; Wang, L.; Bu, F.; Wei. J.; Pan, D.; Wu, M. Hierarchical 3D All-Carbon Composite Structure Modified with N-Doped Graphene Quantum Dots for High-Performance Flexible Supercapacitors. *Small*, **2018**, *14*,1801498.

[14] Deng, X.; Li, J.; Zhu, S.; Ma, L.; Zhao, N. Boosting the capacitive storage performance of MOF-derived carbon frameworks via structural modulation for

supercapacitors. *Energy Storage Materials*, **2019**,*23*, 491-498.
[15] Kale, V. S.; Hwang, M.; Chang, H.; Kang, J.; Chae, I. S.; Jeon, Y.; Yang, J.; Kim, J.; Ko, Y. J.; Piao, Y.; Hyeon, T. Microporosity-Controlled Synthesis of Heteroatom Codoped Carbon Nanocages by Wrap-Bake-Sublime Approach for Flexible All-Solid-State-Supercapacitors. *Advanced Functional Materials*, **2018**, *28*, 1803786.
[16] Peng, Z.; Hu, Y.; Wang, J.; Liu, S.; Li, C.; Jiang, Q.; Lu, J.; Zeng, X.; Peng, P.; Li, F. F. Fullerene-Based In Situ Doping of N and Fe into a 3D Cross-Like Hierarchical Carbon Composite for High-Performance Supercapacitors. *Advanced Energy Materials*, **2019**, *9*, 1802928.
[17] Peng, H.; Yao, B.; Wie, X.; Liu, T.; Kou, T.; Xiao, P.; Zhang, Y.; Li, Y. Pore and Heteroatom Engineered Carbon Foams for Supercapacitors. *Advanced Energy Materials*, **2019**, *9*, 1803665.
[18] Cao, F.; Zhao, M.; Yu, Y.; Chen, B.; Huang, Y.; Yang, J.; Cao, X.; Lu, Q.; Zhang, X.; Zhang, Z.; Tan, C.; Zhang, H. Synthesis of Two-Dimensional $CoS_{1.097}$/Nitrogen-Doped Carbon Nanocomposites Using Metal-Organic Framework Nanosheets as Precursors for Supercapacitor Application. *Journal of the American Chemical Society*, **2016**, *138*, 6924-6927.
[19] Chen, L. F.; Zhang, X. D.; Liang, H. W.; Kong, M.; Guan, Q. F.; Chen, P.; Wu, Z. Y.; Yu, S. H. Synthesis of nitrogen-doped porous carbon nanofibers as an efficient electrode material for supercapacitors. *Acs Nano*, **2012**, 6, 7092-7102.
[20] Li, H.; Li, J.; Thomas, A.; Liao, Y. Ultra-High Surface Area Nitrogen-Doped Carbon Aerogels Derived From a Schiff-Base Porous Organic Polymer Aerogel for CO_2 Storage. *Advanced Functional Materials*, **2019**, *28*, 1904785.
[21] Chen, C.; Yu, D.; Zhao, G.; Du, B.; Tang, W.; Sun, L; Sun, Y.; Besenbacher, F.; Yu, M. Three-dimensional scaffolding framework of porous carbon nanosheets derived from plant wastes for high-performance supercapacitors. *Nano Energy*, **2016**, *27*, 377-389.
[22] Yao, L.; Wu, Q.; Zhang, P.; Zhang, J.; Wang, D.; Li, Y.; Ren, X.; Mi, H.; Deng,

L.; Zheng, Z. Scalable 2D Hierarchical Porous Carbon Nanosheets for Flexible Supercapacitors with Ultrahigh Energy Density. *Advanced Materials*, **2018**, *30*, 1706054.

[23] Yuksel, R.; Buyukcakir, O.; Panda, P. K.; Lee, S. H.; Jiang, Y.; Singh, D.; Hansen, S.; Adelung, R.; Mishra, Y. K.; Ahujia, R.; Ruoff, R. S. Necklace-like Nitrogen-Doped Tubular Carbon 3D Frameworks for Electrochemical Energy Storage. *Advanced Functional Materials*, **2020**, *30*, 1909725.

[24] Li, Y.; Zheng, S.; Liu, X.; Li, P.; Sun, L.; Yang, R.; Wang, S.; Wu, Z. S.; Bao, X.; Deng, W. Q. Conductive Microporous Covalent Triazine-Based Framework for High-Performance Electrochemical Capacitive Energy Storage. *Angewandte Chemie International Edition*, **2018**, *57*, 7992-7996.

[25] Ding, C.; Liu, T.; Yan, X.; Huang, L.; Ryu, S.; Lan, J.; Yu, Y.; Zhong, W. H.; Yang, X. An ultra-microporous carbon material boosting integrated capacitance for cellulose-based supercapacitors. *Nano-Micro Letters*, **2020**, *12*, 1-17.
